# *X-Ray Calc 3*: improved software for simulation and inverse problem solving for X-ray reflectivity

**DOI:** 10.1107/S1600576724001031

**Published:** 2024-03-15

**Authors:** Oleksiy V. Penkov, Mingfeng Li, Said Mikki, Alexander Devizenko, Ihor Kopylets

**Affiliations:** a ZJU-UIUC Institute, Zhejiang University, Haining, Zhejiang 314400, People’s Republic of China; b Department of Mechanical Science and Engineering, University of Illinois Urbana-Champaign, Urbana, IL 61801, USA; c Department of Electrical and Computer Engineering, University of Illinois Urbana-Champaign, Urbana, IL 61801, USA; d National Technical University Kharkiv Polytechnic Institute, Kharkiv 61002, Ukraine; SLAC National Accelerator Laboratory, Menlo Park, USA

**Keywords:** computer simulations, X-ray reflectivity, fitting, *X-Ray Calc 3*, inverse problems, Levy flight particle swarm optimization, structure reconstruction, periodic multilayer structures

## Abstract

This work introduces *X-Ray Calc* (*XRC*), an open-source software package designed to simulate X-ray reflectivity (XRR) and address the inverse problem of reconstructing film structures on the basis of measured XRR curves. *XRC* features a user-friendly graphical interface that facilitates interactive simulation and reconstruction. The software employs a recursive approach based on the Fresnel equations to calculate XRR and incorporates specialized tools for modeling periodic multilayer structures. This article presents the latest version of the *X-Ray Calc* software (*XRC3*), with notable improvements. These enhancements encompass an automatic fitting capability for XRR curves utilizing a modified flight particle swarm optimization algorithm. A novel cost function was also developed specifically for fitting XRR curves of periodic structures. Furthermore, the overall user experience has been enhanced by developing a new single-window interface.

## Abstract 2

This work introduces *X-Ray Calc 3*, an open-source software package for simulating X-ray reflectivity (XRR) and reconstructing film structures on the basis of measured XRR curves, featuring a user-friendly interface and improved fitting capabilities.

## 1. Introduction

X-ray reflectivity (XRR) has emerged as a prevalent technique in analyzing thin-layered materials, encompassing semiconductor heterostructures, metallic multilayers and various thin-film systems. The precise determination of crucial structural parameters, such as interface roughness, density and thickness, plays a pivotal role in comprehending the other properties exhibited by the sample (Björck & Andersson, 2007 ▸; Penkov *et al.*, 2020 ▸). Nevertheless, reconstructing the intricate structure of multiple layers through XRR curves entails a non-linear inverse problem that necessitates computationally intensive calculations. The forward problem encompasses the propagation of waves, involving both reflection and transmission, through the successive layers. This recursive process is highly sensitive to minute intricacies such as wave polarization (Björck & Andersson, 2007 ▸). To tackle the forward problem of XRR, Parratt’s exact recursive method (Parratt, 1954 ▸) is employed for precise computation.

Conversely, the inverse problem necessitates implementing global optimization techniques. However, these methodologies encounter analogous challenges associated with computationally demanding ill-posed non-linear inverse modeling problems. Although the inverse problem poses a considerable difficulty, automating the extraction process of structure parameters for periodic multilayer mirrors (PMMs) using XRR measurements is imperative, as manual curve fitting becomes impractical when confronted with complex PMM configurations.

XRR has become a standard instrument in laboratories developing X-ray optics. Over the years, various commercial and open-source software packages have been developed to simulate XRR. These include *GenX* (Björck & Andersson, 2007 ▸), *JGIXA* (Ingerle *et al.*, 2016 ▸), *REFLEX* (Vignaud & Gibaud, 2019 ▸), *Multifitting* (Svechnikov, 2020 ▸), *XOP* (Río & Dejus, 2011 ▸) and *X-Ray Calc 2* (Penkov *et al.*, 2020 ▸). One of the earliest software packages, *IMD*, was introduced by Windt (1998 ▸) for modeling and analyzing multilayer films. *X-Ray Calc* (*XRC*) emerged as a user-friendly and efficient alternative to *IMD*. Its initial version was released in 2002, followed by significant upgrades in 2016 and 2020 (Penkov *et al.*, 2020 ▸). The primary focus of the previous versions of *XRC* was to manually fit simulated and measured XRR curves of PMMs. Despite its nearly 20-year existence, *XRC* continues to be widely utilized in numerous laboratories (Voronov *et al.*, 2006 ▸; Yamada *et al.*, 2023 ▸; Wu *et al.*, 2021 ▸; Broekhuijsen, 2021 ▸).

However, as previously mentioned, solving the inverse problem in XRR analysis on the basis of measured data requires tackling a challenging task that involves numerous non-linear optimization parameters. A recent study demonstrated that implementing the Levy flight particle swarm optimization (LFPSO) algorithm can greatly enhance the solution of the inverse XRR problem (Li *et al.*, 2023 ▸). The Levy flight is a form of random walk that employs the Levy distribution to determine the size of each step taken. This search strategy is renowned for its efficiency in global optimization, owing to the long jumps made by the particles (Haklı & Uğuz, 2014 ▸; Jensi & Jiji, 2016 ▸). Initially, the LFPSO algorithm was implemented by combining MATLAB with a parallelized command-line version of *XRC* (Li *et al.*, 2023 ▸). The MATLAB code generated a set of solutions (particles) and invoked the *XRC* executable to calculate the XRR for each solution. The ‘cost function’ was used to evaluate the solutions. The cost function was the chi-squared (χ^2^) goodness-of-fit test, a type of Pearson chi-squared test. We use it to test whether the fitted XRR curve differs from the target (experimental) curve. The best solution and the corresponding value of the cost function were then returned. Through iterative repetition of this process, it was observed that ∼80% of the computation time was spent on data exchange between MATLAB and *XRC*. Consequently, it is anticipated that the computation time can be further reduced by directly incorporating the LFPSO algorithm into *XRC*.

In this research article, we introduce the latest iteration of *X-Ray Calc* software, denoted as *XRC3* (major version 3), which incorporates automated fitting of XRR data and facilitates the reconstruction of coating structures, thus addressing the inverse problem. Significant enhancements have also been made to the fitting of periodic structures. Moreover, the initial version of the LFPSO algorithm has been refined to enhance convergence and a novel cost function for fitting XRR curves of periodic structures has been devised. Additionally, the associated graphical user interface (GUI) has undergone substantial improvements to enhance the overall user experience.

## 2. Algorithms

This section briefly describes the XRR computation algorithm and mainly focuses on the automatic fitting of XRR curves.

### 2.1. X-ray reflectivity

The algorithms used in *XRC* for calculating XRR are described in detail elsewhere (Penkov *et al.*, 2020 ▸; Li *et al.*, 2023 ▸). The Parratt algorithm is used for simulating specular XRR. A Gaussian distribution is assumed for roughness. The refractive indices of materials are calculated using the Henke tables for XRR (Henke *et al.*, 1993 ▸). The implementation of the computational algorithm has been validated against the *IMD* software (Windt, 1998 ▸). XRR curves for various single, few and periodic multilayer structures were simulated using both software packages, utilizing the same database of X-ray scattering factors in both instances. A point-by-point comparison of the results revealed minimal divergence between *XRC* and *IMD* results, with accumulated variances of the whole XRR curve consistently below 0.5%. Any observed distinctions between these programs could reasonably be ascribed to the accumulation of rounding errors. This validation process collectively underscores *XRC*’s precision and accuracy.

### 2.2. Coating structure representation and fitting modes

An effective representation of a coating’s structure plays a pivotal role in enhancing user experience and computational effectiveness. Various methods for representing the fitted structure have been integrated into the software to better align with practical requirements. Throughout both the *XRC* software and this article, the following terminology is employed to elucidate the description of a coating structure intended for simulation or fitting:

(*a*) Fitting – the process of solving inverse problems, which can be carried out manually or automatically. Manual fitting was introduced in the previous versions of *XRC* (Penkov *et al.*, 2020 ▸). Automatic fitting is described in the present article.

(*b*) Material – the chemical composition of a layer, for example, Mo or MoB_2_. This value is used to find the values of atomic scattering factors, atomic weights and table densities in the embedded materials database. It cannot be changed during fitting.

(*c*) Layer – the basic ‘geometrical’ entity. The layer consists of a material and has three parameters: thickness, roughness of the top surface and density. All three parameters will be subject to adjustment during the fitting procedure.

(*d*) Stack – a set of layers. It can be single or periodic.

(*e*) Structure – a set of stacks, including a ‘substrate’. The substrate is a special type of stack consisting of a single layer with infinite thickness.

**Figure 1:**
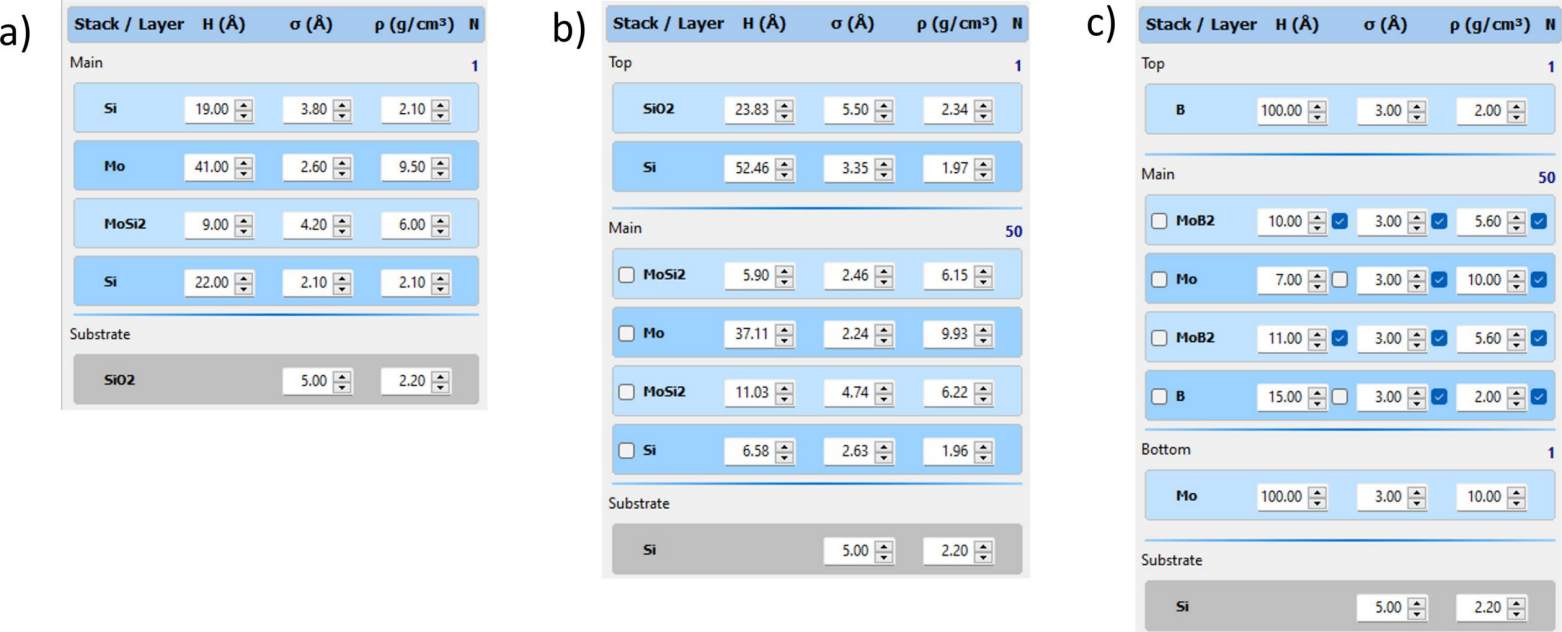
Examples of different types of fitting structures. (*a*) Regular structure. (*b*) Periodic structure. (*c*) Periodic structure with distributions: the thicknesses of the Mo and Si layers are varied across the stack; the rest of the parameters are paired.

For example, Fig. 1 ▸(*a*) illustrates a structure comprising a single stack, with the substrate included. Figs. 1 ▸(*b*) and 1 ▸(*c*) demonstrate more complicated structures. In Fig. 1 ▸(*b*), the stack named ‘Top’ is a surface layer consisting of SiO_2_ and Si. ‘Main’ is periodic, comprising four layers repeated 50 times. The ‘Bottom’ stack in Fig. 1 ▸(*c*) represents a Mo sublayer. Additional examples of diverse structures are presented in Fig. S1 of the supporting information. The general types are:

(i) Regular structure – every parameter of each layer is treated independently. This mode is used for non-periodic coatings consisting of several layers. An example of such a structure is shown in Fig. 1 ▸(*a*).

(ii) Periodic structure – the coating consists of repeated stacks; every stack consists of several layers [Fig. 1 ▸(*b*)].

(iii) Periodic structure with distributions – in this context, ‘distribution’ refers to utilizing nonuniform layer thicknesses. In such instances, additional guidelines can be implemented to depict the distribution of a specific parameter across the periodic stack.

In the previous implementation of the LFPSO algorithm (Li *et al.*, 2023 ▸), each layer in the structure was treated independently, which was suitable for non-periodic coatings (referred to as the ‘regular structure’). However, this approach inefficiently utilizes computational resources when fitting periodic structures like regular PMMs. For instance, consider a typical Mo/Si PMM as a relatively simple model with one periodic stack in the middle and single-surface and sublayer stacks. If the level of periodicity is high, the stack can be represented using a set of coupled parameters. For example, the thickness of a specific layer (Mo or Si) is the same across all stacks.

Consequently, the total number of variables is significantly reduced. For instance, if the aforementioned Mo/Si PMM contains 60 repeated stacks, the total number of variables is reduced from 2 × 3 + 60 × 4 × 3 = 726 (using the regular approach) to 2 × 3 + 1 × 4 × 3 = 18. This reduction in the number of variables is substantial. Generally, a structure can comprise single and periodic stacks [*e.g.* as shown in Figs. 1 ▸(*b*) and  ▸(*c*)], and each stack is treated independently.

‘Distributions’ provide a convenient means to describe coatings with periodically stacked layers that exhibit stochastic variations in the periodicity parameter *D*. Such coatings may have a graded period, as seen in supermirrors, or the periodicity may be distorted due to unstable deposition conditions, among other possibilities. In a general case, each parameter within the entire coating can be treated independently.

At the start of the fitting procedure, the software expands each periodic stack into individual layers. For example, a Mo/Si PMM with 60 repeated stacks will be transformed into a structure comprising a single stack consisting of 60 layers of Mo alternated with 60 layers of Si. Subsequently, this structure will be fitted as a regular stack. Once the fitting is complete, the software generates a distribution chart for each parameter of every material type (*e.g.* thickness, roughness, density) within the structure. This feature allows for a comprehensive understanding of the parameter distributions across the coating.

Typically, independently treating every parameter of each layer is not physically meaningful. For example, while the thickness of a certain layer may vary during the deposition process due to an unstable deposition rate, the roughness and density of each layer type remain constant across the whole multilayer stack. The software enables the ‘stack locking’ of specific parameters in such cases. An example of such a structure is illustrated in Fig. 1 ▸(*c*), where the expected variation occurs in the thickness of Mo and B from the top to the bottom of the coating while other parameters are paired. After fitting, distribution charts will only be generated for non-paired parameters. This approach allows for a more focused analysis of the parameters that exhibit variations, providing valuable insights into the specific aspects of the coating’s properties.

In many practical situations, the arbitrary distributions of the structure’s parameters across the stack can also be meaningless. If the deposition rate changes due to variations in deposition conditions, this process will lead to gradual changes in thickness rather than arbitrary oscillations. Usually, such changes in thickness can be described as polynomial functions such as

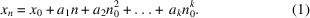

Thus, the number of fitting parameters can be further reduced. For example, 60 different layer thicknesses of Si from the above example can be replaced with only three or four parameters: the thickness of the first layer (*x*_0_) and two or three coefficients of the polynomial function (*a*_1_–*a*_*k*_). The user can select the order of the polynomial function from 1 to 20.

The result of such a fitting is shown in Fig. S2. Overall, the software provides various methods for describing the fitted structure, as shown in Table 1 ▸, and users can choose the most suitable depending on the nature of their coatings.

### 2.3. Fitting algorithms

**Figure 2:**
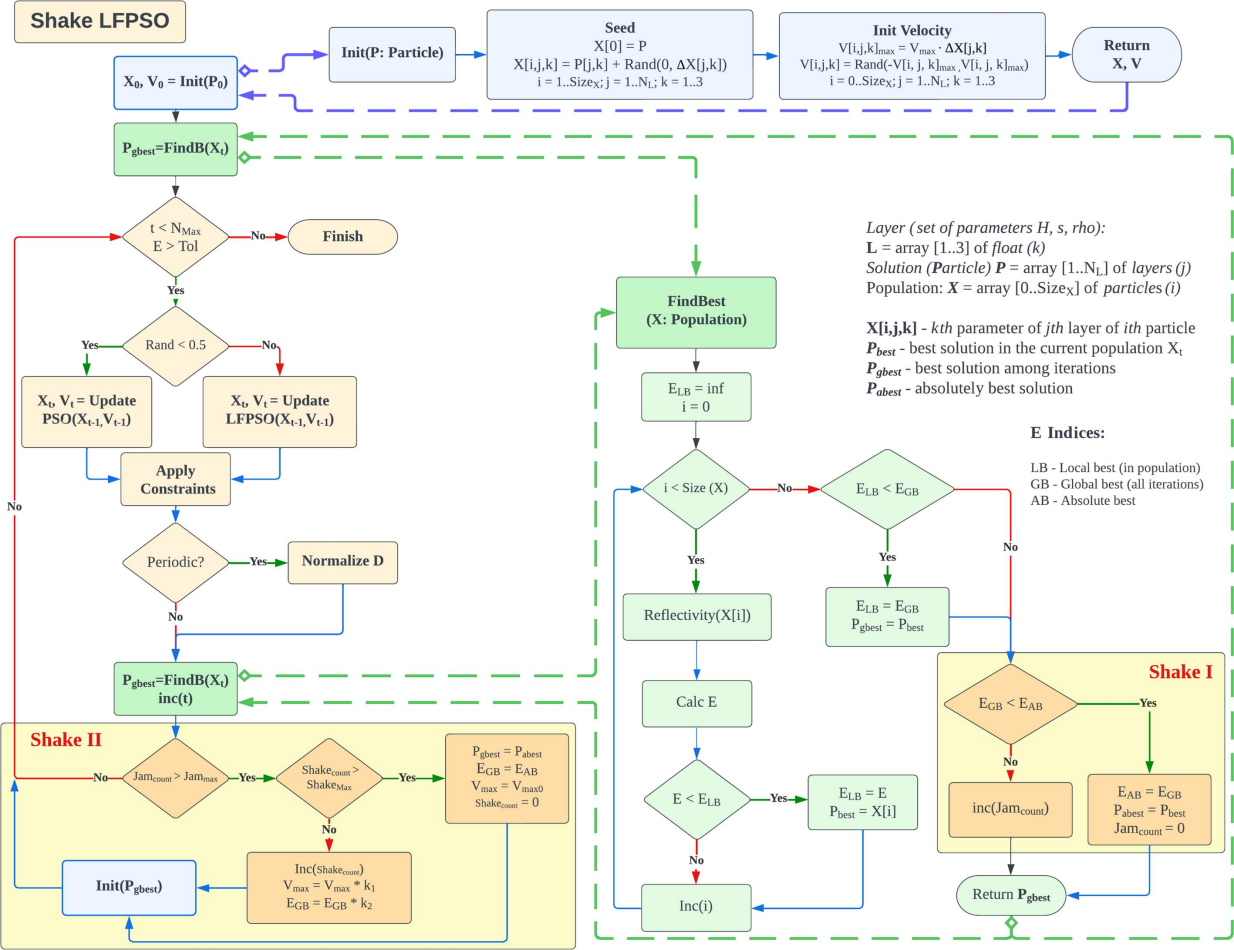
A general diagram of the Shake LFPSO algorithm. Bold dashed lines indicate call of subroutines.

The general flow of the algorithm called ‘Shake LFPSO’ implemented for the automatic XRR curve fitting is shown in Fig. 2 ▸. This is a further development of the LFPSO algorithm reported earlier (Li *et al.*, 2023 ▸). The LFPSO algorithm, like most evolutionary algorithms, involves creating a population of solutions (particles), which is a vector of parameters. This population is then modified according to a specific scheme to generate new individuals better suited to the problem at hand than the previous population. This process is known as a generation, and it is repeated until a stopping criterion is met or the maximum number of iterations is reached (Kennedy *et al.*, 2001 ▸). The population is a three-dimensional array *X*, in which the first dimension represents various solutions, and its length is the size of the population (Size_*X*_) defined by the user. The second dimension represents all layers in the model, so its size *N*_L_ equals the number of layers, including the substrate. The last dimension represents the fitted parameters of every layer (thickness, roughness, density), so its size is 3.

The algorithm comprises several subroutines (Init, FindBest) distinguished by different colors. The invocation of these subroutines is denoted by dashed bold lines (Fig. 2 ▸). The roles and functions of these subroutines are elucidated below.

#### 2.3.1. Initialization

The software proposes two methods for the seeding of the initial population. The first method (SeedR) can be used when the initially calculated XRR curve differs greatly from the experimental curve. For instance, only the sequence of layers is known, and all parameters can vary widely. In such cases, the user is requested to define thickness, toughness and density ranges. Then, the initial population is seeded as follows:

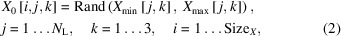

where *X*_min_ and *X*_max_ are low and high constraints, respectively. Rand is a function returning a random value in the range indicated.

When the initially calculated XRR curve closely aligns with the experimental curve, or when the subsequent fitting aims to refine the previous fitting results, it is sensible to initialize the initial population, denoted as *P*_0_, on the basis of the best-known solution. The initial population *X*_0_ is seeded in the function Init. The function accepts a solution *P* as the input parameter and returns a new population *X* as follows:

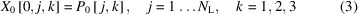

and

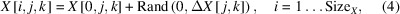

where Δ*X*[ *j*, *k*] contains ranges for each of the structure’s parameters as defined by the user. Consequently, each solution within the population adheres to the same set of boundary constraints. These constraints can be generated automatically prior to the fitting procedure or manually specified through the user interface, thus ensuring physically meaningful parameter ranges. LFPSO also necessitates the initialization of particle velocities within a swarm. In the present implementation, the velocity for each parameter is established as follows. First, the matrix of maximum velocities is calculated:

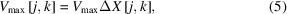

where *V*_max_ is a constant user-defined parameter. The initial velocities are set as follows:

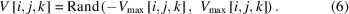

After seeding the initial population and velocities according to equations (3)–(6), the best solution for the current population is checked, and the best solution, *P*_gbest_, is found using the cost function described in this article.

Additionally, the periodicity parameter of a stack, *D*, representing the cumulative thickness of individual layers within a given period, is computed during the initialization stage. The calculation of the *D* value for each periodic stack is carried out as follows:

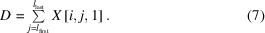

Here, *l*_first_ and *l*_last_ are the current stack’s first and last layers. This value is stored in the internal data structure for further use.

#### 2.3.2. The main loop

The PSO/LFPSO iteration is executed as depicted in functions ‘Update PSO’ and ‘Update LFPSO’ in Fig. 2 ▸. Within the PSO iteration, both the population *X* and the velocities *V* are updated, leveraging the information from the best solution obtained thus far, denoted as *P*_gbest_, as described in the previous subsection. Subsequently, boundary constraints are applied to each element *X*[*i*, *j*, *k*] and *V*[*i*, *j*, *k*]. The next step involves the normalization of periodic stacks, as elaborated in Section 2.3.3. Following the constraint and normalization of the population, the new best solution *P*_gbest_ is determined. The iteration loop persists until the cost function attains its minimum or the maximum number of iterations is reached. These parameters are user defined, affording control over the algorithm’s tolerance and the total computation time.

#### 2.3.3. Periodicity keeper

If a certain stack in the structure is periodic, then the thickness of every layer is normalized to maintain the total period of the stack *D*. The normalization is performed as follows:

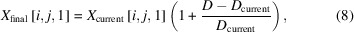

where *D*_current_ is the periodicity parameter for every periodic stack in the current solution. It is calculated using equation (7).

#### 2.3.4. Finding the best solution

An XRR curve is simulated for every solution (particle) in the population, and the deviation between the calculated and target curves is calculated using the following equation:

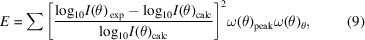

where *I*(θ)_exp_ and *I*(θ)_calc_ are the measured (experimental) and computed intensity of XRR for every angle θ, ω(θ)_peak_ is the peak weight function, and ω(θ)_θ_ is the incidence-angle weight function.

**Figure 3:**
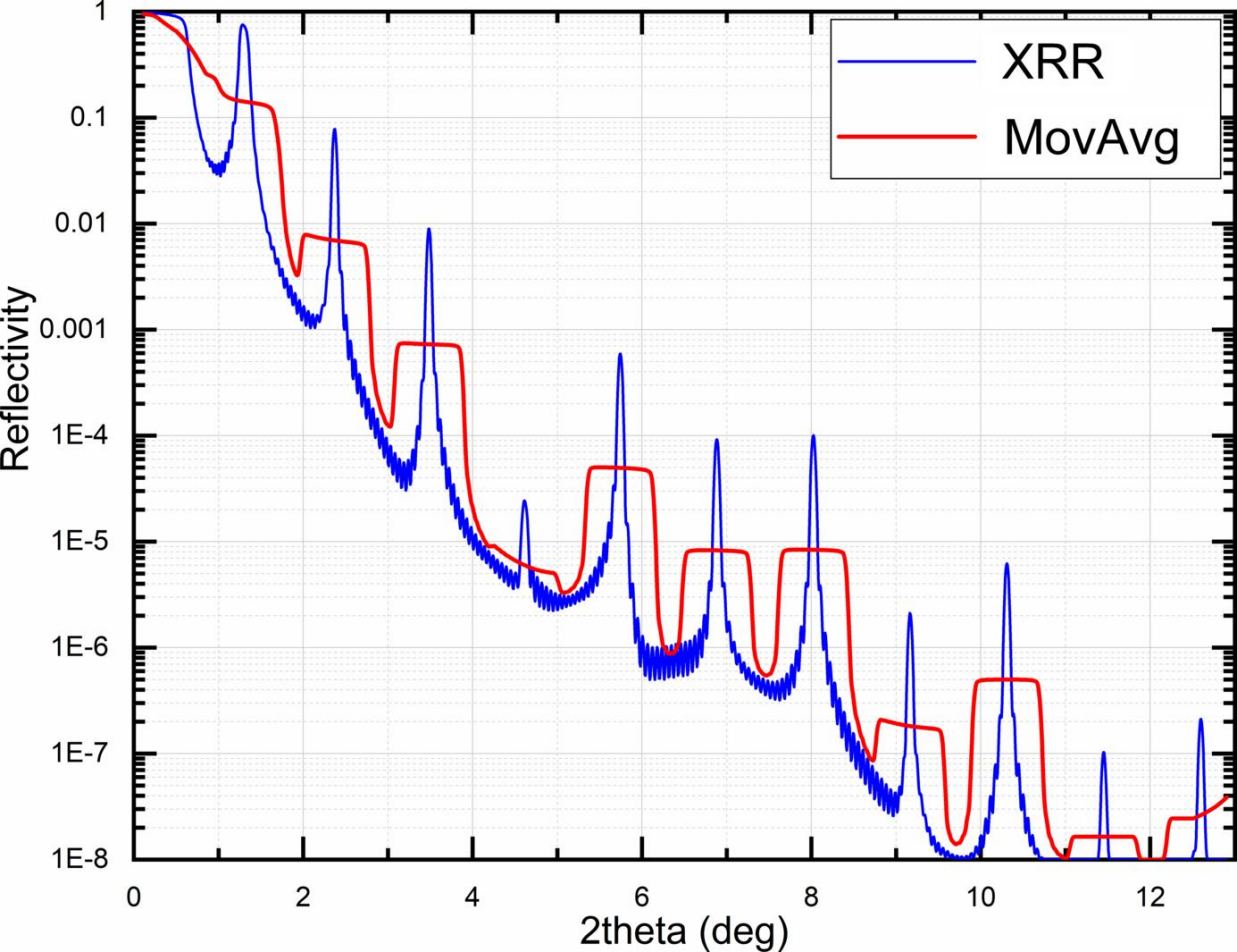
The XRR curve and corresponding moving average used to define the peak weight function.

The peak weight function helps work with the XRR curves of PMMs. In such cases, parameters of the periodic stack affect the intensity of primary diffraction peaks; Kiessig fringes are affected by the substrate and surface layers. Implementing the peak-weight function allows focus on fitting the primary features of the XRR curve. For applying the peak weight function, the primary diffraction peaks should be found. The fastest way is to compute the moving average function with a relatively large window, as shown in Fig. 3 ▸. Then, for every theta, the weight function is calculated as follows:

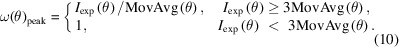

Using the peak weight function is optional; it is enabled in the fitting control panel. The incidence-angle weight function is also optional. It enables concentration of the fitting process on distinct segments of the XRR curve, or, in other words, it allows one to specify which part of the XRR curve is more important for the fitting. The function is calculated as follows:

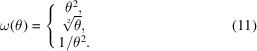

The user can select any of these functions or choose to use ω(θ)_θ_ = 1 (no weight, default value).

#### 2.3.5. Shake LFPSO

**Figure 4:**
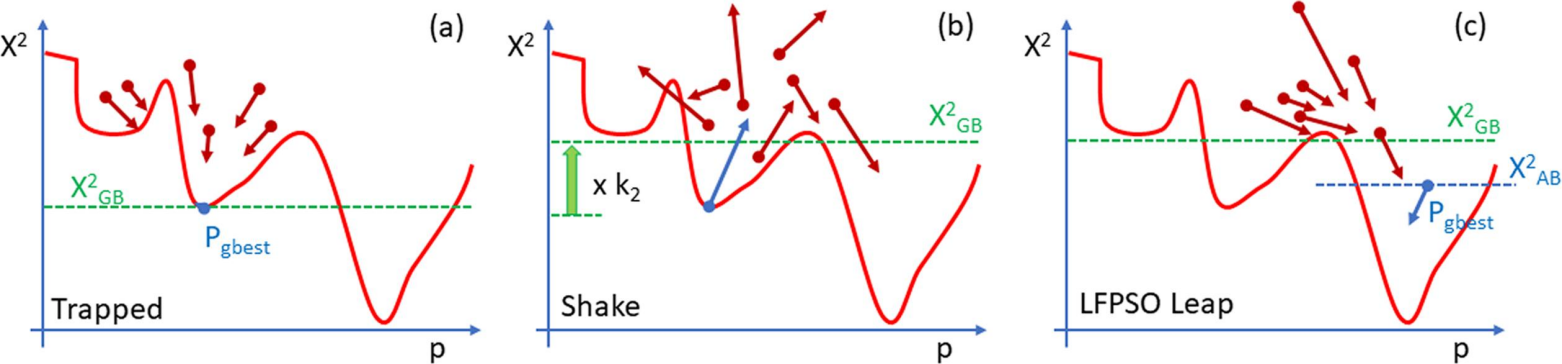
The idea of the Shake LFPSO algorithm. The red line represents a cost function in a single dimension. Dark red arrows indicate location and velocity vectors of particles (solutions) in the population *X* *_t_*. (*a*) Shake condition: all particles are trapped in a local minimum. (*b*) Shaking event: location and velocities of all particles are randomized on the basis of *P*_gbest_. The maximum of the velocities is multiplied by *k*1, while 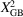 is lifted by a factor *k*_2_. (*c*) After LFPSO leap, the best new solution *P*_gbest_ is found.

To enhance the convergence of the LFPSO algorithm when applied to the inverse XRR problem, a novel modification has been introduced. The concept behind this modification is to navigate the system out of a local minimum, as illustrated in Fig. 4 ▸. The condition for triggering this action is based on the number of iterations that have transpired without reducing the cost function. This parameter can also be configured via the GUI. The ‘Shake’ functionality is divided into two segments, denoted as ‘Shake I’ and ‘Shake II’ in Fig. 2 ▸.

Block I is called at the end of the subroutine finding the best solution in the current population, FindBest. If the absolute lowest value of the cost function *E*_AB_ is not improved, the counter Jam_count_ increases by 1. Otherwise, the counter is reset to 0, *E*_GB_ is assigned to *E*_AB_, and the best solution in the current population, *P*_best_, is assigned to the absolute best solution *P*_abest_.

Block II is called at the end of the main loop if the condition for Shake is satisfied (Jam_count_ is larger than a user-defined threshold). In this block, the Shake event is performed. This event can have several iterations. First, the number of continuous Shake iterations is checked. If it does not exceed the allowed maximum, the Shake iteration is performed as follows. (1) Coefficient *V*_max_ [equation (5)] is multiplied by the factor *k*_1_. (2) The stored value of the lowest achieved global cost function *E*_GB_ is multiplied by the factor *k*_2_. (3) The counter of Shake iterations is increased by 1. (4) Then the Init subroutine (Section 2.3.1) is called, using the best solution *P*_gbest_ as the initial solution.

Parameters *k*_1_ and *k*_2_ could be considered as additional heating leading to a temperature rise in traditional simulated annealing optimization algorithms. The user defines the values of factors *k*_1_ and *k*_2_, which determine the maximum allowable Shake iterations via the GUI. If the number of consecutive Shake iterations surpasses the predefined maximum limit, the most recent Shake event is deemed unsuccessful, and the system reverts to its previous state.

## 3. Software description

**Figure 5:**
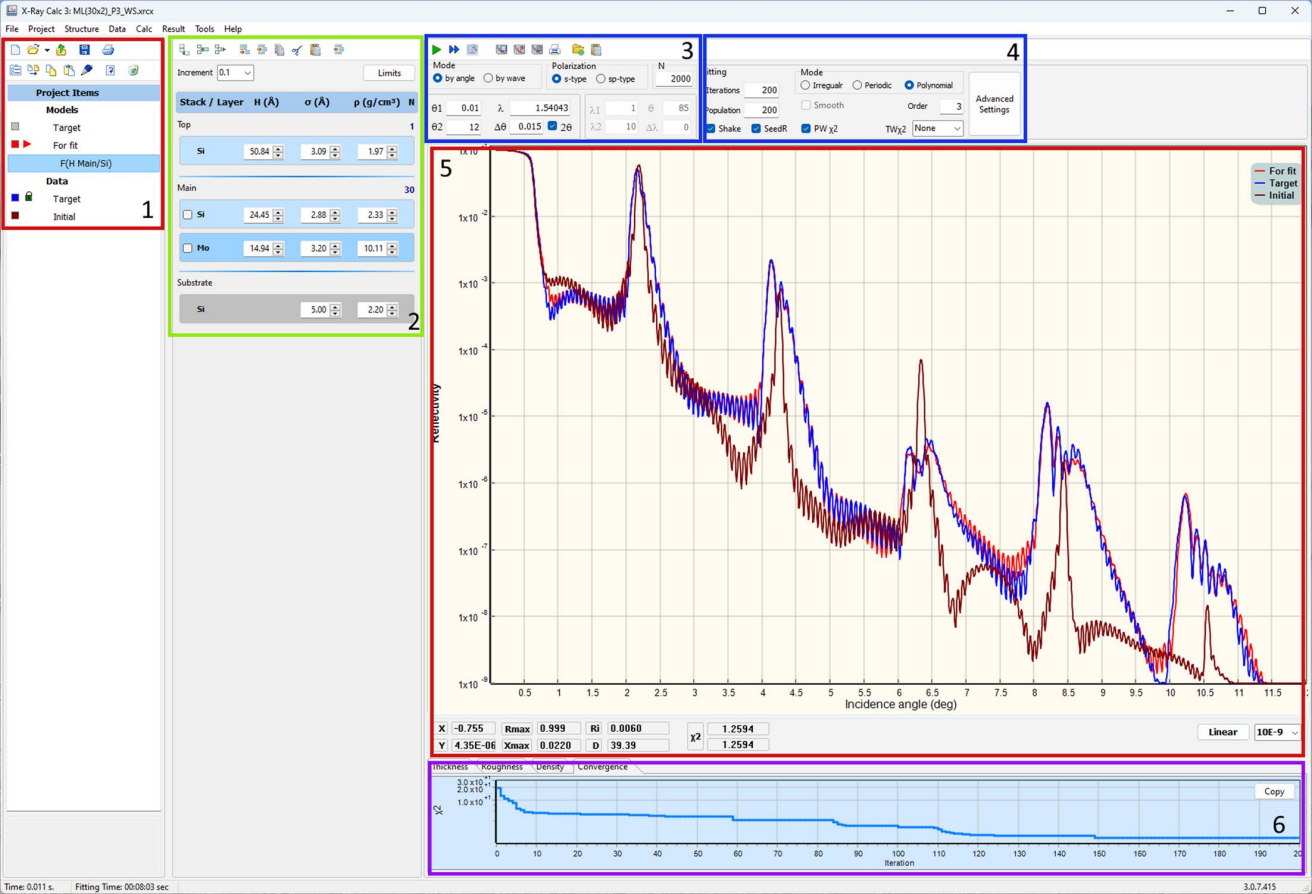
A screenshot of the GUI for *XRC3*. (1) Project tree. (2) Structure panel. (3) XRR calculation parameters. (4) Fitting parameters. (5) XRR curves. (6) Distributions/Convergence panel.

The software was developed using Embarcadero Delphi 11.3, a high-level programming language. It is distributed as a standalone executable (.exe) file. The software features a user-friendly interface designed for working with scattering structure models as well as calculated and experimental XRR curves (see Fig. 5 ▸ for reference). Compared with earlier versions, the interface has undergone a comprehensive redesign, providing users with quick and convenient access to core functions through a unified single-window interface. The primary window comprises four key panels. The first panel, labeled ‘Project Items’, showcases the contents of the current project. A project, contained within a single file, can organize an unlimited number of theoretical models and experimental curves into well structured folders. The software supports concurrent computation of XRR curves for each model within the project. The ‘Project Items’ panel is equipped with various tool buttons that facilitate straightforward manipulation of project items, including actions like duplication, copy/paste and more.

The structure panel functions as a visual depiction of the layered model and provides easy access to all parameters linked with the structural model. The software empowers users with full control over computation parameters, including settings for wavelength, the number of points in computed curves, grazing-angle range and instrumental beam divergence. Reflectivity calculations can be performed as functions of either incident angle or wavelength, offering flexibility to suit user preferences. The main plot allows for the overlay of multiple computed and experimental curves, enabling users to compare the degree of matching visually. The vertical scale can be switched between linear and logarithmic for convenience, and users can zoom in on individual regions of the plot.

Layer controls allow editing of every parameter in the structure. Also, parameters can be reduced/increased using spinners. Thus, the software provides quick access to the parameters of the structure, allowing users to manually fit the computed curve to the experimental curve within the same window. The calculation result can be exported as an ASCII or graphic file. Experimental data can be loaded to the project from an ASCII file or pasted from the clipboard. *XRC* provides necessary tools for the experimental data, such as sorting, smoothing and normalization.

Apart from providing a concise representation of periodic structures, the software also offers specialized extensions for modeling, including distribution functions. These extensions enable a gradual variation of specific model parameters, such as thickness, within any periodic stack. This feature proves useful in simulating physical phenomena such as changes in deposition rate during coating manufacturing or the introduction of increased interface roughness due to columnar growth. Additional extensions include tabulated parameter distributions and specialized functions for interface roughness, such as linear, exponential or step functions.

A parallel computation was implemented to enhance the performance of computation and optimization (fitting). The complete angular data range, denoted as *M*, is divided into *n* segments. The value of *n* is then set to match the number of available central processing unit (CPU) cores on the computer platform. For instance, with a CPU containing 16 cores, the typical simulation time for XRR ranges from 0.05 to 0.25 s for a periodic multilayer model consisting of 300 stacks, each comprising four layers. Consequently, the resulting XRR curve consists of 3000 points. Computation times for some typical CPUs are shown in Table S2 of the supporting information. The remarkable computational speed empowers users to perform manual curve fitting in real time, facilitating quick and efficient analysis of experimental data. In addition to improved manual fitting, a new automatic solver based on the modified LFPSO algorithm was implemented, as described in the following sections.

## 4. Performance evaluation

The performance of the fitting algorithm was evaluated on the basis of the convergence as a function of the number of iterations when an ‘unknown’ structure was fitted to a calculated curve. Thus, the target cost function was zero. Table 2 ▸ briefly describes the structures used in the benchmark.

**Figure 6:**
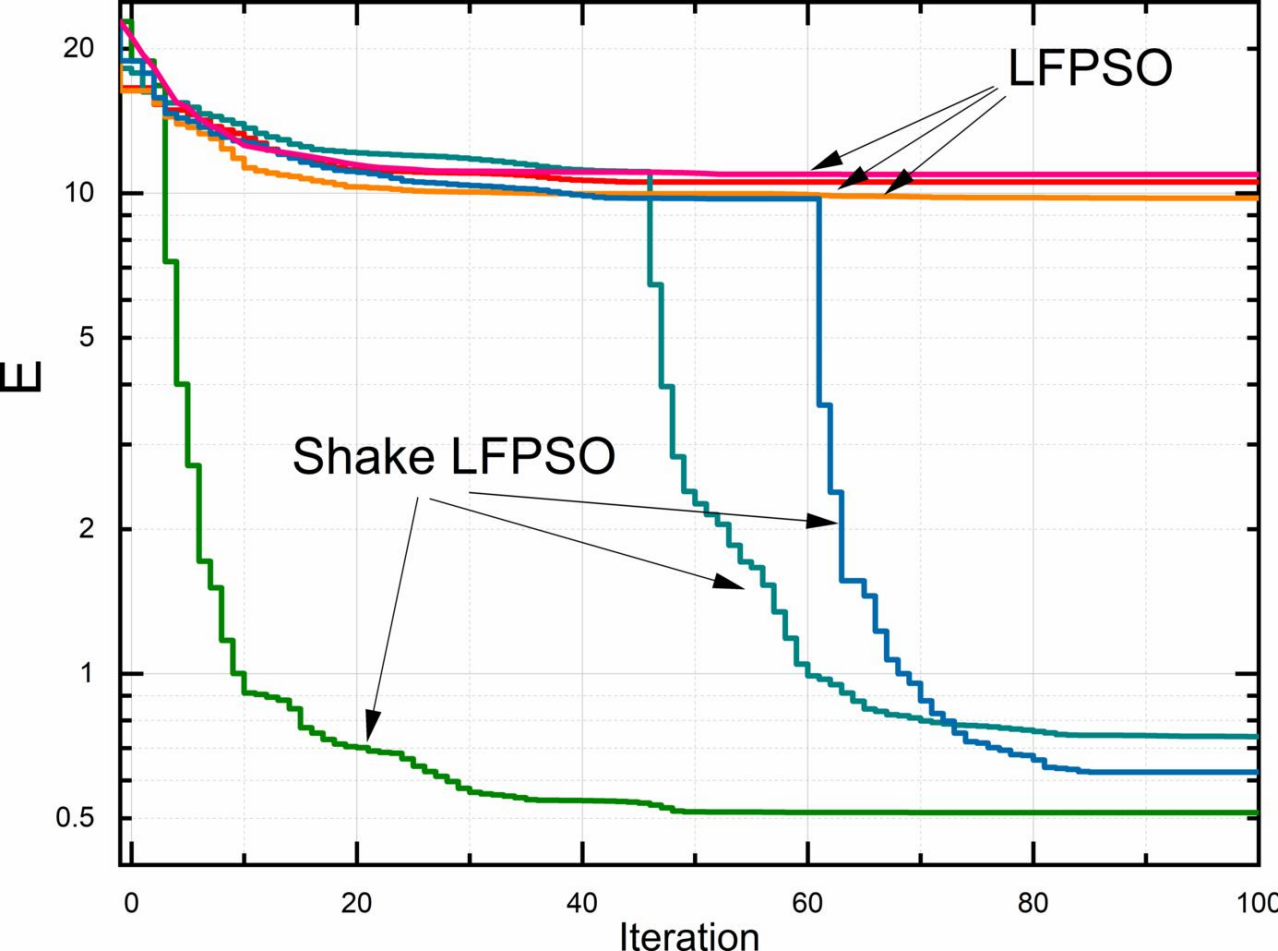
Reduction of the cost function with increasing number of iterations for LFPSO and the Shake LFPSO algorithm (Model: ML 30x2 P3).

Fig. 6 ▸ demonstrates the performance comparison between the ‘classical’ LFPSO and one of the Shake LFPSO algorithms introduced earlier. The calculation was performed for the model shown in Fig. S2. The computation in each mode was repeated three times for consistency and reliability.

The algorithm can be customized for different fitted models by fine-tuning the parameters mentioned above (referred to as ‘magic numbers’) and enabling/disabling various options. To demonstrate the impact of these magic numbers, we benchmarked different combinations of them, as outlined in Table 3 ▸, while using the same initial model (ML 30x2 P3).

The initial value of *E* was ∼25. While pure LFPSO was performed, *E* reduced to ∼10 after 30–40 iterations and remained unchanged with increasing iterations, indicating trapping in a local minimum. In the presence of the Shake mode, the cost function experienced a sharp decline after shaking at the 20th, 45th and 61st iterations for the bright-green, dark-green and blue curves in Fig. 6 ▸, respectively.

**Figure 7:**
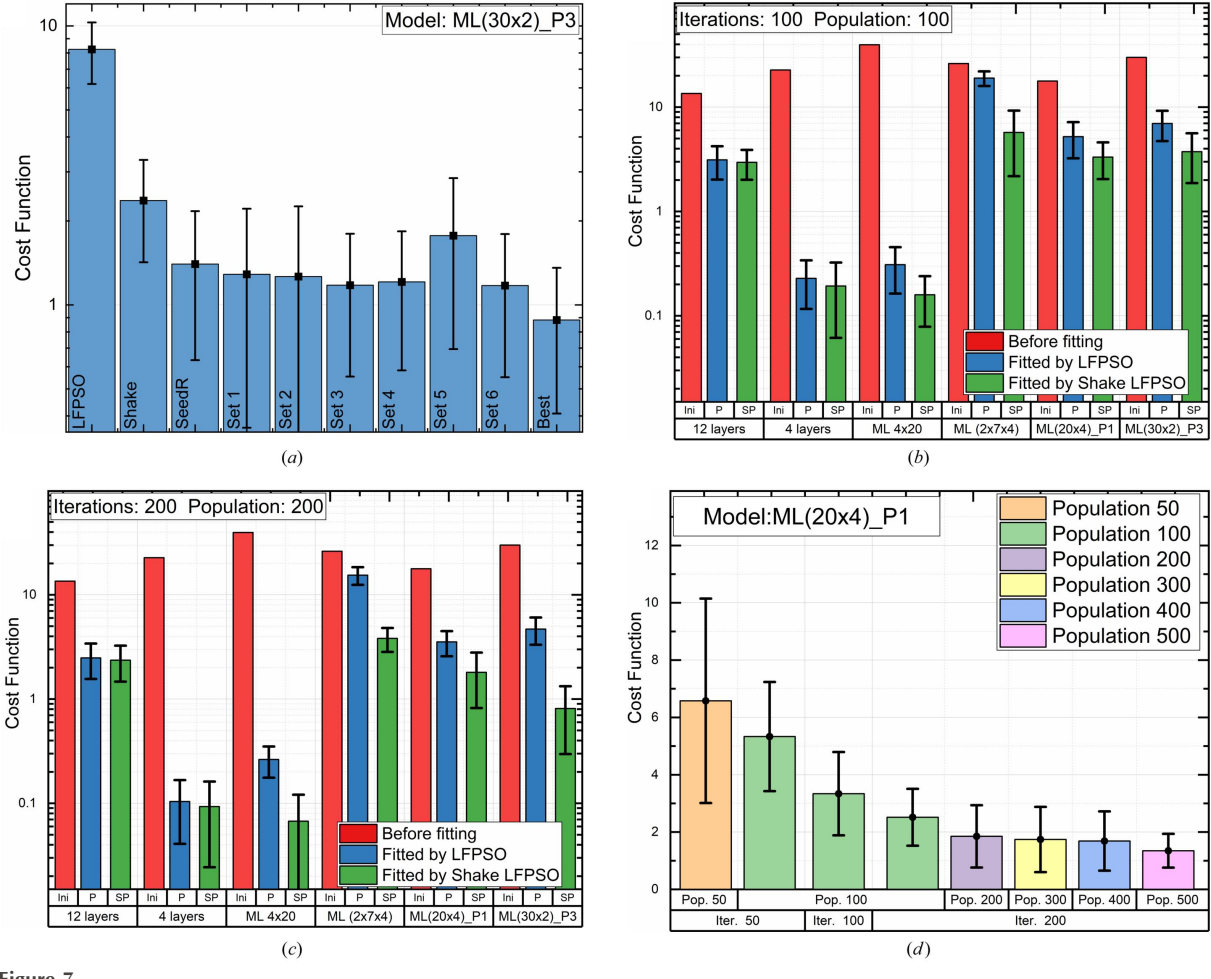
Benchmarking of the algorithm. (*a*) Effect of the algorithm’s modifications (Table 3 ▸). (*b*), (*c*) Comparison of LFPSO and Shake LFPSO for different types of models. (*d*) Effect of the number of iterations and the size of the population on convergence of Shake LFPSO for the given model.

Fig. 7 ▸(*a*) illustrates the effect of the improvements of the LFPSO reported above on the example of model ML 30x2 P3 (Table 2 ▸). Table 3 ▸ shows combinations of the parameters and Fig. 7 ▸(*a*) shows the fitting results with these combinations for the given model. The calculation was performed 20 times for every set of parameters and statistics were calculated. Error bars in Figs. 7 ▸(*a*)–7 ▸(*d*) represent the standard deviation between these 20 runs.

Overall, the effectiveness of the shaking event depends on the structure’s complexity. Figs. 7 ▸(*b*) and 7 ▸(*c*) illustrate the effect of the improvements of the LFPSO on the examples of different types of models. The number of iterations and the population size used was 100 for the first batch [Fig. 7 ▸(*b*)] and 200 for the second batch [Fig. 7 ▸(*c*)]. In Fig. 7 ▸, ‘Ini’ denotes the initial value of the cost function before the optimization, and ‘P’ and ‘SP’ denote the resulting cost function after fitting using classical LFPSO and Shake LFPSO, respectively. Enabling Shake mode improves the convergence for all models; the most significant improvement was achieved for complicated cases: several periodic stacks (ML 2x7x4) and non-linearly graded thickness (ML 30x2 P3).

For the same initial model and set of magic numbers, the final convergence depends on the number of iterations and the size of the population. The computation time linearly increases with the increase of both parameters. As can be seen from Fig. 7 ▸(*d*), it is beneficial to limit the number of iterations to 200 and increase the size of the population. The maximum size is 20 000–30 000 for the 32 bit version of the software. For the 64 bit version, the size of the population is limited by the available memory (RAM).

## 5. Illustrative examples

### 5.1. Single-layer coating

**Figure 8:**
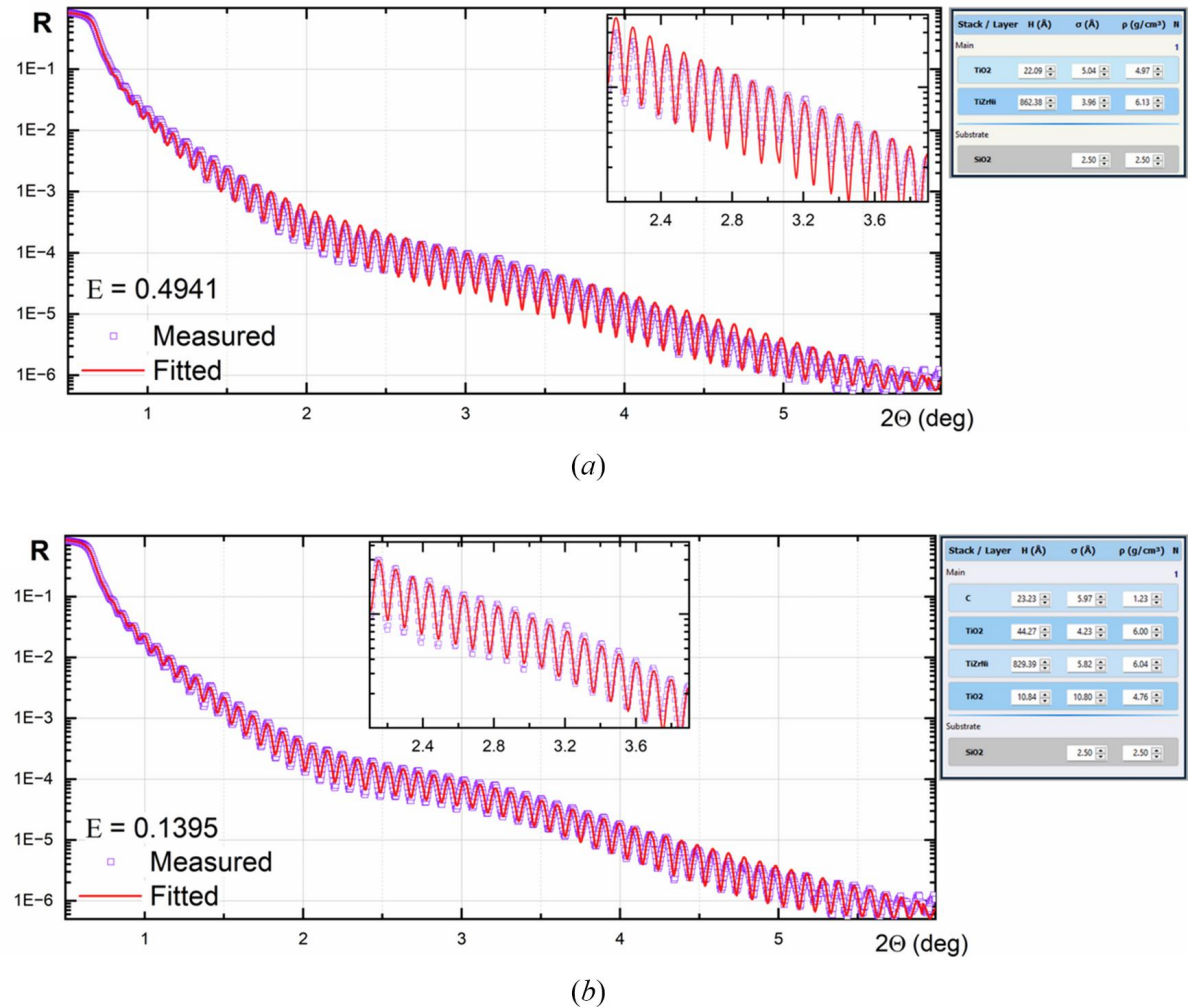
XRR curve fitting for a single-layer coating. Insets show the fitted structures. (*a*) Two-layer model. (*b*) Four-layer model.

This example illustrates the evaluation of a single-layer TiZrNi coating deposited by DC magnetron sputtering onto a glass substrate, as described by Penkov *et al.* (2022 ▸). The XRR was measured using a DRON-3M diffractometer (BOUREVESTNIK, JSC) with filtered Cu *K*α radiation. The measured and best-fitted curves are shown in Fig. 8 ▸. Initially, the fitted model consisted of two layers: TiZrNi with TiO_2_ on top of it. For such a model, the lowest cost-function value was ∼0.48, and visual comparison of the measured and fitted curves shows a significant difference when the diffraction angle is above 3°. Adding an extra TiO_2_ layer beneath and covering carbon-based layers minimizes the cost function to 0.1395 [Fig. 8 ▸(*b*)]. The final structure is shown in the corresponding inset.

Interestingly, the top and bottom TiO_2_ layers had different densities. The density of the bottom layer was 4.76 g cm^−3^, which was ∼10% higher than the reference data (4.26 g cm^−3^). The top layer had a density of 6 g cm^−3^. This indicates the non-stoichiometric composition of the layer and the presence of some heavy atoms (Ni and/or Zr). The low density of the top ‘carbon’ layer indicates that it consists of hydro­carbons, *e.g.* the surface was contaminated with adsorbed oils.

### 5.2. Periodic X-ray mirrors

**Figure 9:**
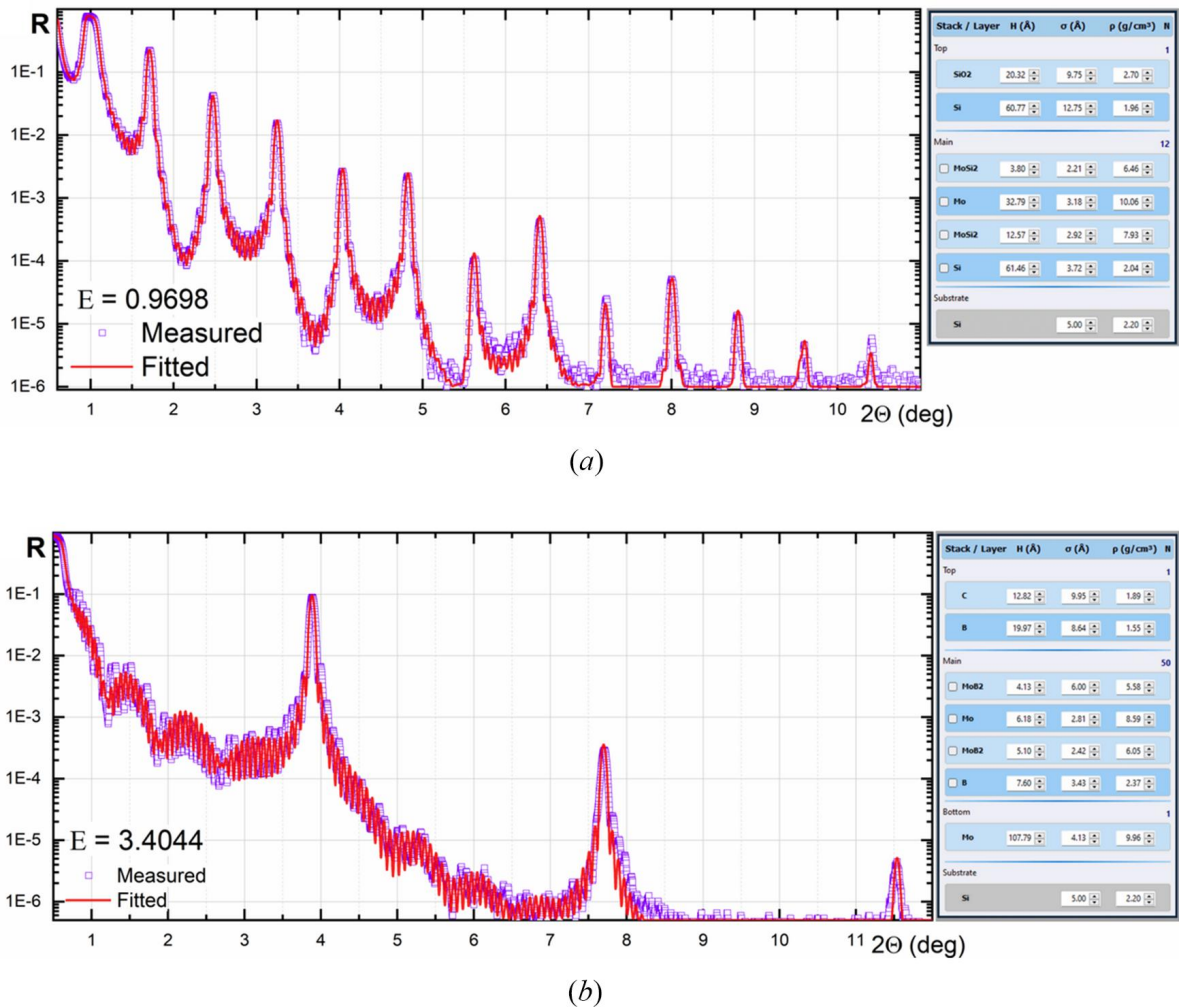
XRR curve fitting for X-ray mirrors. (*a*) Mo/Si. (*b*) Mo/B.

The first example illustrates the fitting of the XRR curve for Mo/Si PMMs deposited by DC magnetron sputtering onto a silicon substrate, as described by Penkov *et al.* (2022 ▸) [Fig. 9 ▸(*a*)]. The XRR was measured using a DRON-3M diffractometer (BOUREVESTNIK, JSC) with filtered Cu *K*α radiation. The fitting was performed in the ‘periodic mode’ (Section 2.3.3). The results revealed well known features of Mo/Si PMMs, such as asymmetrical intermixed zones between Mo and Si.

The second example is a fitting of the XRR curve for Mo/B PMMs with a 2.3 nm period [Fig. 9 ▸(*b*)]. These PMMs were deposited using pulse DC sputtering for Mo and RF sputtering for B as described by Li *et al.* (2023 ▸) and Penkov *et al.* (2021 ▸). The substrate holder was water cooled during the deposition, so the temperature did not exceed 50°C. The XRR was measured using a Malvern Panalytical Empyrean diffractometer equipped with a W/Si Göbel mirror in monochromatic Cu *K*α_1_ radiation.

The XRR curve fitting unveiled the presence of relatively thin and somewhat asymmetric intermixing zones between Mo and B. A prior prediction by Penkov *et al.* (2021 ▸) had anticipated a reduction in intermixing at lower substrate temperatures. Additionally, the fitting brought to light a disparity in Mo density between the periodic structure and the Mo sublayer. The Mo sublayer, which is nearly 10 nm thick, exhibits polycrystalline characteristics, with a density closely resembling that of bulk Mo. In contrast, the density of the 0.6 nm thick Mo layers within the periodic structures was determined to be 84% in comparison with the bulk material.

### 5.3. Polynomial fitting on graded PMMs

**Figure 10:**
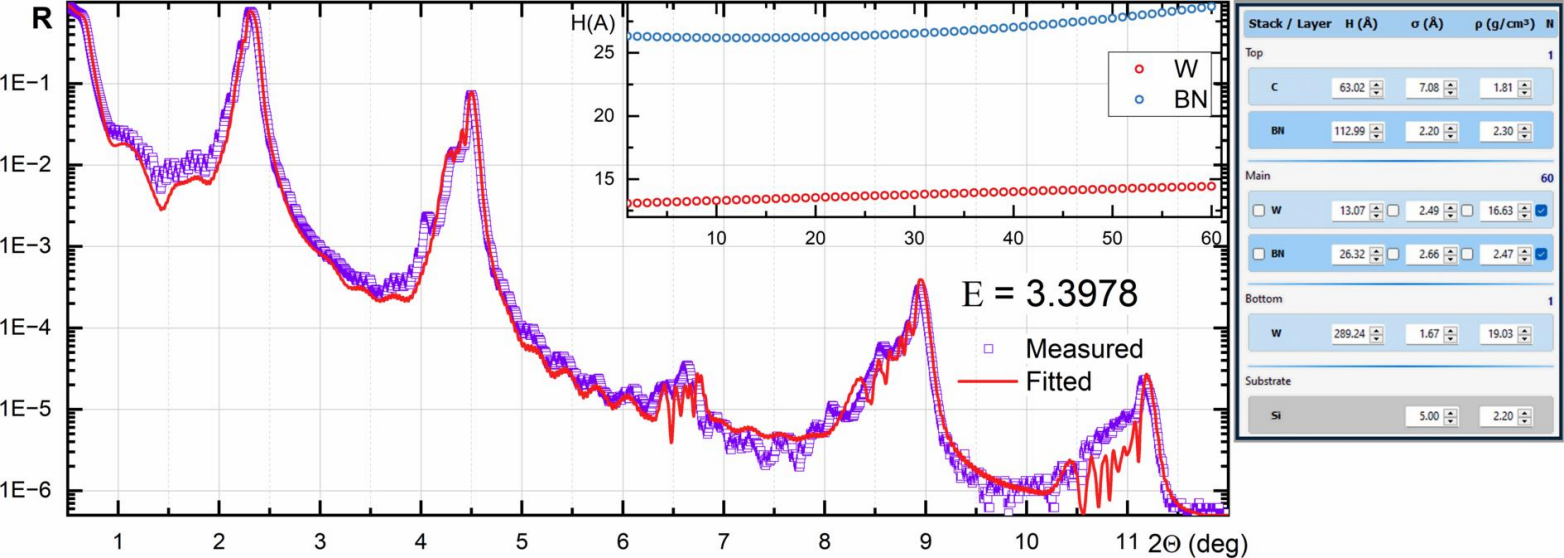
Polynomial XRR curve fitting for a W/BN X-ray mirror with gradually changed thickness of layers.

A W/BN PMM was deposited using pulse DC sputtering for W and RF sputtering for BN as described by Li *et al.* (2023 ▸). The XRR was measured using a Malvern Panalytical Empyrean diffractometer equipped with a W/Si Göbel mirror in monochromatic Cu *K*α_1_ radiation. The XRR curve and the fitting result are shown in Fig. 10 ▸. The broadening and changing of the shape of the diffraction peaks indicate the variation of layer parameters across the stack. The XRR was fitted in polynomial mode. Due to unstable deposition conditions, the thickness and roughness of the layers were found to vary across the multilayer stack, as shown in the inset.

### 5.4. Supermirror

**Figure 11:**
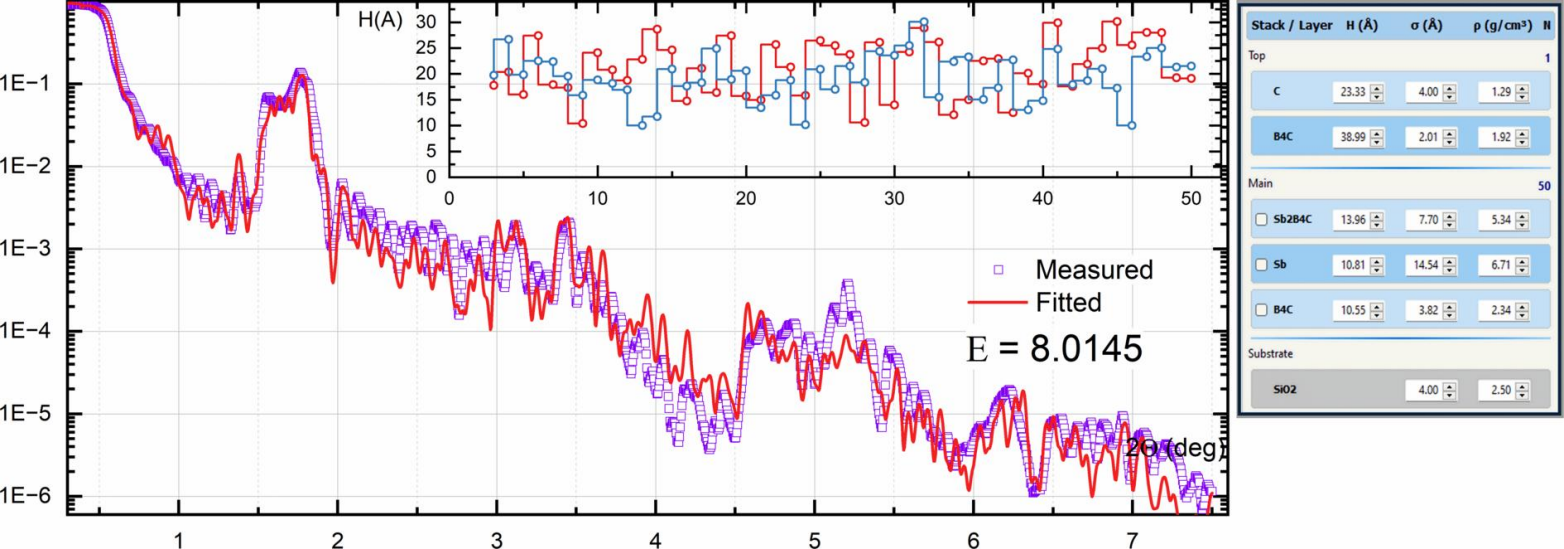
General XRR curve fitting for an Sb/B4C X-ray supermirror. The inset shows reconstructed distributions of layer thicknesses.

An Sb/B_4_C supermirror was deposited by DC magnetron sputtering onto a glass substrate, as described by Vishnyakov *et al.* (2018 ▸). The XRR was measured using a DRON-3M diffractometer with filtered Cu *K*α radiation. The XRR curve (Fig. 11 ▸) was fitted using the non-periodic mode as follows. It was assumed that the density and roughness of similar layers and the thickness of the intermixed zone did not change along the multilayer stack, so these parameters were marked as ‘linked’. Thus, only the thicknesses of the Sb and B_4_C layers varied. The calculated XRR curve represented the main key features of the measured curve; the inset in Fig. 11 ▸ shows the reconstructed thickness distribution for these layers, which correlated with the expected thickness distribution given the exposure time of these layers during manufacturing.

## 6. Conclusions

This article has presented the latest enhanced iteration of the *X-Ray Calc* software, specifically developed for XRR curve simulation and fitting. *X-Ray Calc* is an open-source software designed to operate seamlessly on Windows platforms. It boasts easy installation, rapid calculation capabilities and a user-friendly interface. The software has incorporated a highly efficient XRR curve fitting algorithm rooted in the LFPSO method. The new algorithm significantly reduces the computational time compared with other global optimization methods such as genetic algorithms.

## 7. Program distribution and technical details

The software was developed using the programming language Delphi under Windows. The authors provide it for free under the GNU General Public License. The package includes executables, a database of optical constants and sample files for fitting. The user manual for the software is available online. Pre-compiled executables, source code and other resources are available through a GIT repository at the following links:

(*a*) GIT repository – https://github.com/OleksiyPenkov/X-RayCalc3.

(*b*) Online manuals, examples and benchmarks used in this article – https://github.com/OleksiyPenkov/X-RayCalc3/wiki.

Delphi free community edition or any Delphi *RAD Studio* commercial edition is required to modify and build executables using the source code. Using the most recent version of Embarcadero Delphi (free community edition or commercial license) is recommended. The following free open-source libraries should be installed in Delphi:

(i) FastMM5 – https://github.com/pleriche/FastMM5.

(ii) VirtualTreeView – https://github.com/TurboPack/VirtualTreeView.

(iii) FastMath – https://github.com/neslib/FastMath.

(iv) OmniThread Library – https://github.com/gabr42/OmniThreadLibrary/tree/release-3.07.7.

## Supplementary Material

Supporting information. DOI: 10.1107/S1600576724001031/te5127sup1.pdf

## Figures and Tables

**Table 1:** Summary of structure-representation methods and their applicability

Structure representation	Applicability	Computation time
General	Regular non-periodic coatings	Long
Periodic	PMMs	Short
Periodic with arbitrary deviations	Supermirrors, PMMs with arbitrary parameter distributions	Long
Polynomial distributions	PMMs with gradual change of parameters	Moderate

**Table 2:** Description of structures used for benchmarking

Structure	Type	Description
4 layers	Regular	4 layers [Fig. 1 ▸(*a*)]
12 layers	Regular	12 layers
ML 4x20	Periodic	1 stack, 4 layers, 20 periods
ML 2x7x4	Periodic	2 stacks, 4 layers, and 7 periods each
ML 20x4 P1	Periodic	1 stack, 4 layers, 20 periods [Fig. 1 ▸(*c*)], linearly graded thickness for the Si layer
ML 30x2 P3	Periodic	1 stack, 2 layers, 30 periods [Fig. 1 ▸(*c*)], hyperbolically graded thickness for the Si layer

**Table 3:** Combinations of parameters used for benchmarking [Fig. 7 ▸(*a*)] Bold type indicates a change compared with the previous line in the table.

	Parameter						
Name of setup	Shake	SeedR	*V*_max_	ω_1_	ω_2_	*k*_1_	*k*_2_
LFPSO	Off	Off	N/A	N/A	N/A	N/A	N/A
Shake	**On**	Off	0.1	0.1	0	1	1
Set 1	On	**On**	**0.3**	**0.3**	0	1	1
Set 2	On	On	0.3	0.3	**0.1**	1	1
Set 3	On	On	0.3	0.3	0.1	**2**	1
Set 4	On	On	0.3	0.3	0.1	2	**2**
Set 5	On	On	0.3	0.3	0.1	**3**	1
Set 6	On	On	0.3	0.3	0.1	3	**2**
Best	On	On	0.3	0.3	0.1	**1.41**	**1.41**
