# Supplementary material for: X-Ray Calc 3: improved software for simulation and inverse problem solving for X-ray reflectivity
Source: J Appl Crystallogr. 2024 Mar 15;57(Pt 2):555–66. doi: 10.1107/S1600576724001031 (PMC11001402; doi:10.1107/S1600576724001031)
Supplement: Supplementary file 1 [file j-57-00555-sup1.pdf]

JOURNAL OF APPLIED CRYSTALLOGRAPHY

Volume 57 (2024)

Supporting information for article:

X-Ray Calc 3: improved software for simulation and inverse problem solving for X-ray reflectivity

Oleksiy V. Penkov, Mingfeng Li, Said Mikki, Alexander Devizenko and Ihor Kopylets

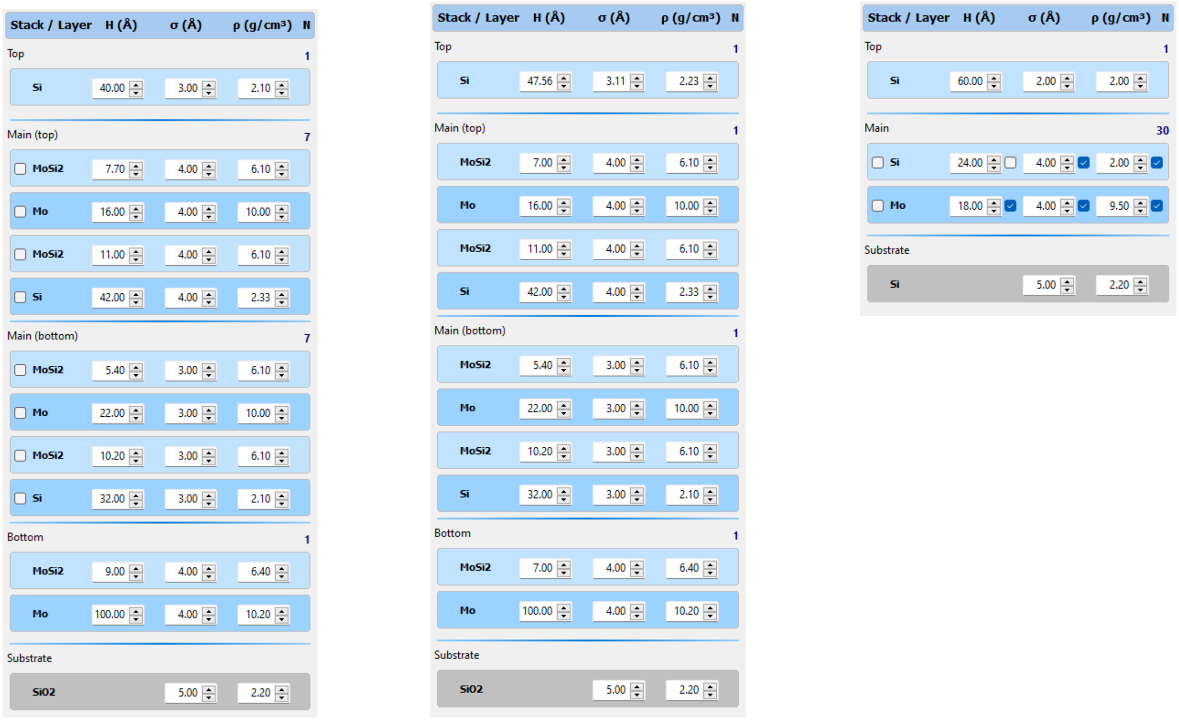

Figure S1 Examples of fitted structures. a) Multilayer coating with two periodical stacks (N = 2x7). b) non-periodical structure, 12 layers in total. c) Simple periodical structure (N=30) with a top layer.

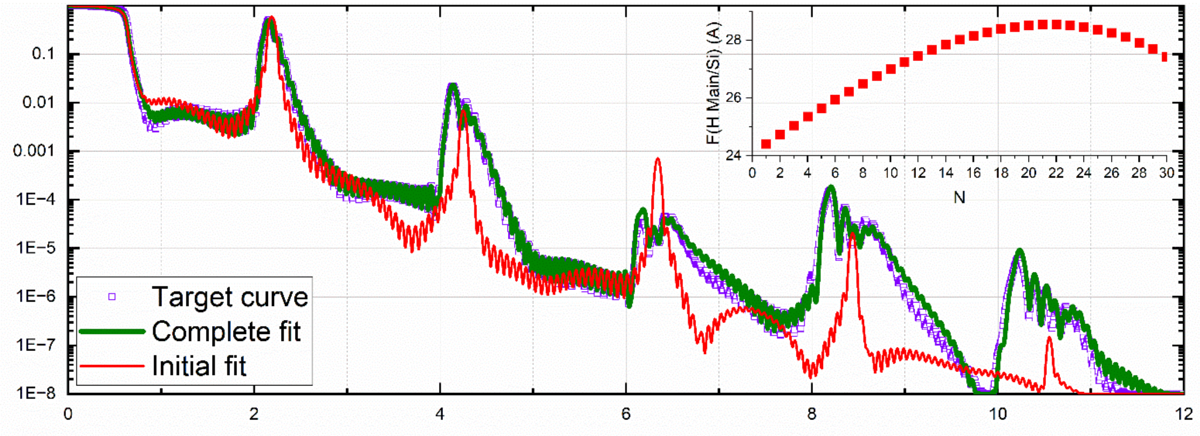

Figure S2 XRR fitting for test structure ML30x2 (P3). Inset shows reconstructed distribution of thickness of Si layer.

Table S1 Computation time of XRR curves (θ-2θ mode, 2000 points) on various CPUs.

CPU	CPU Cores/Threads	Computing time (sec)			
		4 Layers	12 Layers	Multilayer 30x2 (P3)	Multilayer (300x4)
Intel Core i7-12700H	14/20	0.006	0.007	0.011	0.090
Intel Core i9-10900K	10/20	0.003	0.003	0.007	0.057
Intel Core i9-13900KF	24/32	0.002	0.002	0.014	0.065
AMD Ryzen 9 3950X	16/32	0.005	0.006	0.018	0.065

Table S2 Solving of optimization problem on various CPUs. Default settings. Size: 200, Population: 200.

CPU	CPU Cores/Threads	Structure Model			
		4 Layers	12 Layers	Multilayer 30x2 (P3)	Multilayer (300x4)
Intel Core i7-12700H	14/20	1 min 57 sec	2 min 13 sec	5 min 32 sec	75 min
Intel Core i9-10900K	10/20	1 min 18 sec	2 min 00 sec	3 min 35 sec	41 min
Intel Core i9-13900KF	24/32	1 min 32 sec	1 min 37 sec	3 min 59 sec	44 min
Intel Core i9-13900KF	8/16*	1 min 07 sec	1 min 18 sec	3 min 4 sec	40 min
AMD Ryzen 9 3950X	16/32	4 min 23 sec	4 min 23 sec	7 min 06 sec	52 min
